# Routine immunohistochemistry study for polyomavirus BK nephropathy in transplanted kidney biopsies, is it recommended?

**DOI:** 10.1186/s12882-021-02444-5

**Published:** 2021-06-18

**Authors:** Fatemeh Nili, Maliheh Mohammadhoseini, Seyed Mohammadreza Khatami, Golnar Seirafi, Majidreza Haghzare

**Affiliations:** 1 Department of Pathology, Imam Khomeini Hospital Complex, Tehran University of Medical Sciences, Tehran, I.R. of Iran; 2 Educated of Tehran University of Medical Sciences, Tehran, I.R. of Iran; 3 Department of Nephrology, Tehran Heart Center, Tehran University of Medical Sciences, Tehran, I.R. of Iran

**Keywords:** Polyomavirus BK nephropathy, Diagnosis, Immunohistochemistry, Simvian virus 40

## Abstract

**Background:**

Early diagnosis and treatment of Polyomavirus BK Nephropathy (PVBKN) is a challenging issue in the management of patients with kidney transplantation. Currently, histopathologic diagnosis is the gold standard method for diagnosis of PVBKN. However, typical viral inclusions may not be found in early stages of the PVBKN and should, instead, be diagnosed using immunohistochemistry (IHC) study. There is no clear consensus about routine IHC tests in the pathologic evaluation of transplanted kidney biopsy samples.

**Material and methods:**

The current study was conducted on transplanted kidney biopsy samples, since 2016 to 2019. The patients who have presented with new onset of allograft dysfunction, at least 2 weeks after transplantation surgery, were included in our study. All these biopsy samples were evaluated with routine renal biopsy stains as well as IHC for SV40 (Simvian Virus 40) antigen. The identification of typical nuclear virus inclusion body and any nuclear positive staining on IHC (≥1+ positive result) were considered as definite evidence of PVBKN. Sensitivity, specificity, Positive Predictive and Negative Predictive Values (PPV and NPV) of histopathologic assessment without IHC study were evaluated.

**Results:**

Among 275 included cases, 18 (6.5%) patients with PVBKN were diagnosed. In patients with PVBKN, typical viral inclusions were detected in 14 samples (77.7%), on primary histopathological examination. However, virus-infected cells were identified just after IHC study in 4 (22.2%) of patients. Sensitivity, Specifity, PPV and NPV of morphologic histopathological assay without IHC for detection of PVBKN was 77.7, 100, 100 and 98.4% respectively.

**Conclusion:**

Routine IHC study for SV40 in all transplanted kidney biopsy samples with new onset of allograft dysfunction, will enhance the diagnostic sensitivity of early stage disease detection.

## Background

Polyomavirus BK nephropathy (PVBKN) is an important cause of allograft dysfunction in patients with kidney transplantation [1]. Polyomavirus is a non-enveloped double stranded DNA virus which exists latently in the human body. After primary infection of urothelial cells, virus DNA will be integrated into the host’s genome. The virus adapts with the host and stays in urothelial cells for a long time [1, 2]. After immunosuppression, following transplantation, the virus activates, replicates, and damages urothelial cells as well as renal parenchymal tubules. The presence of the virus in the urine (viruria) is the first step of virus activation that progresses to viremia and nephropathy [1–4].

PVBKN incidence is about 1–10% in different populations [5]. In high-risk graft recipients who have immunologically desensitized, the incidence is higher (20%) than the others [6]. True risk factors related to PVBKN infection are not well known. However, previous studies suggest factors such as older age, male gender, over immunosuppression, the degree of HLA mismatch, prior rejection episodes, urethral stent placement, prolonged cold ischemia, and BK serostatus are associated with PVBKN infection [5, 7–9]. Early detection of the disease allows the clinician to reduce the immunosuppression and the possibility of graft loss in over 80% of the patients [7, 10].

Definitive diagnosis of PVBKN is based on histological findings including the nuclear inclusion body in tubular epithelial or parietal glomerular cells [11]. These viral nuclear inclusion bodies are usually associated with epithelial cell necrosis and acute tubular injury. IHC or In Situ Hybridization (ISH) assays can confirm PVBK infection [12, 13]. Due to focal pattern of PVBKN involvement, at least two biopsy cores, including medullary tissue are needed, to conduct IHC and ISH assays. In 10 to 30% of the patients, sampling error can occur. So a negative result does not exclude PVBKN [11, 12]. On the initial stage of renal infection, characteristic viral inclusion bodies cannot be detected on routine morphologic study. In these cases, IHC study will be helpful. In most of the clinics, antibody for the anti-large T antigen of Simian virus 40 (SV40) is used. However, anti-BKV PV1 and anti-Agnoprotein are also available [14, 15]. There is not any consensus about applying IHC on every biopsy sample. Some authors suggest IHC staining just in the case of high clinical suspicion, including high viremia level, presence of decoy cells on urine cytology or suspicious nuclear changes [14]. On the other hand, some others suggest it as a routine [16]. This study was conducted to evaluate the significance and the importance of routine IHC study in all biopsy samples in patients presenting with allograft dysfunction.

## Material and methods

### Patient selection and follow-up

All the patients who have undergone kidney transplantation in two referral hospitals in Tehran from 2016 to 2019 and have consequently presented with new onset of allograft dysfunction 2 weeks after the transplant surgery, were included in this cross-sectional study. Kidney biopsy specimens, urine, and serum PCR results of the patients were examined. Demographic data, type of the donor, time of transplantation and clinical presentation were asked from the patients or gathered from the pathology request forms. The study was approved in local ethics committee in Tehran University of Medical Sciences. The study protocol is performed in accordance with the relevant guidelines. Informed consent were obtained from the participants.

### Histopathological assays

Biopsy samples received in Buffered Formalin fixative were embedded in paraffin blocks. Serial 2–3 μm thick sections were taken from each block and stained with Hematoxilin & Eosin, PAS, Masson’s Trichrome and Jone’s methods. In addition, IHC study for SV40 antigen (PAb416, Biocare Medical Company: USA) according to the kit protocol was performed on all samples. Beside the viral inclusion body evaluation, other histopathological changes such as interstitial fibrosis (ci), tubular atrophy (ct), tubulitis (t), and total inflammation (ti) were evaluated and categorized based on the last Banff lesion scores classification 2019 [17, 18]. Through immunohistochemistry study, every nuclear staining with SV40 (≥1+ intensity) in tubular epithelial cells was considered as definite PBKVN.

### Statistical analysis

The normality of the distribution of the samples was examined by Kolmogrof-Smirnov test using SPSS software version 23 (IBM Inc., Chicago, Illinois, USA). Qualitative data were compared using Chi-square or Exact Fisher’s test. The *p*-values less than 0.05 were considered statistically significant. Using pathology results and IHC (SV40) as the gold standard diagnostic test, Sensitivity, Specifity, Positive and Negative Predictive Values (PPV and NPV) for H&E morphology without IHC study were calculated using 2× 2 tables (Table 1).

**Table 1:** Number of the patients with and without morphologic evidence of viral inclusion on H&E study and total number of the patients with positive IHC result for SV40

	Positive IHC study	Negative IHC study
Presence of viral inclusions in morphology	14 (true positive cases)	0 (false positive cases)
Absence of inclusions in morphology	4 (false negative cases)	257 (true negative cases)

## Results

Total number of 317 patients were referred to our hospital for histopathological evaluation of kidney biopsy specimens. Forty-two (13%) samples which were small in size, containing less than 10 glomeruli, were excluded because of inadequacy. In 275 included patients, 65.6 and 34.4% were male and female, respectively. Mean and Standard deviation (SD) of the patients age were 45.6 ± 13 (ranging from 16 to 76) year, respectively. The allograft biopsy was taken in a mean period of 40 (ranging from 1 to 360) months after transplantation. Majority of the patients (95.6%) were presented with increased creatinine, and small number with proteinuria and hematuria (4.4%).

The most frequent diagnoses in our patients were acute T-cell mediated rejection (ATCMR) (24%) and chronic active T-cell mediated rejection (17%), followed by Calcineurin Inhibitor (CNI) toxicity (11%), mixed chronic T cell and antibody-mediated rejections (7%), PVBKN (6.5%), chronic active antibody mediated rejection (5%), tubulointerstitial nephritis and fibrosis (NOS) (4%). The other miscellaneous diagnoses had lower frequencies.

The total of 18 patients with PVBKN were diagnosed, of which 72% were male and 28% were female. In patients without PBKN, 65%, were male and 35% were female. There was no statistical difference in sex predilection between patients with or without PVBKN (*P* = 0.57).

The mean age of patients with PVBKN was 47 ± 13 years. In all patients whose definitive diagnosis was PVBKN, the only clinical sign was elevated serum creatinine (2.93 ± 1.3 mg/dl). PVBKN was diagnosed 23.7 ± 13 (range 6–36) months after kidney transplantation. According to the Banff working group new classification [19], 63.6% of patients with PVBKN were classified as class 2 disease. The rest (36.4%) were equally classified as class 1 and 3. During the follow-up, 38.5% of the grafts were lost.

**Fig. 1:**
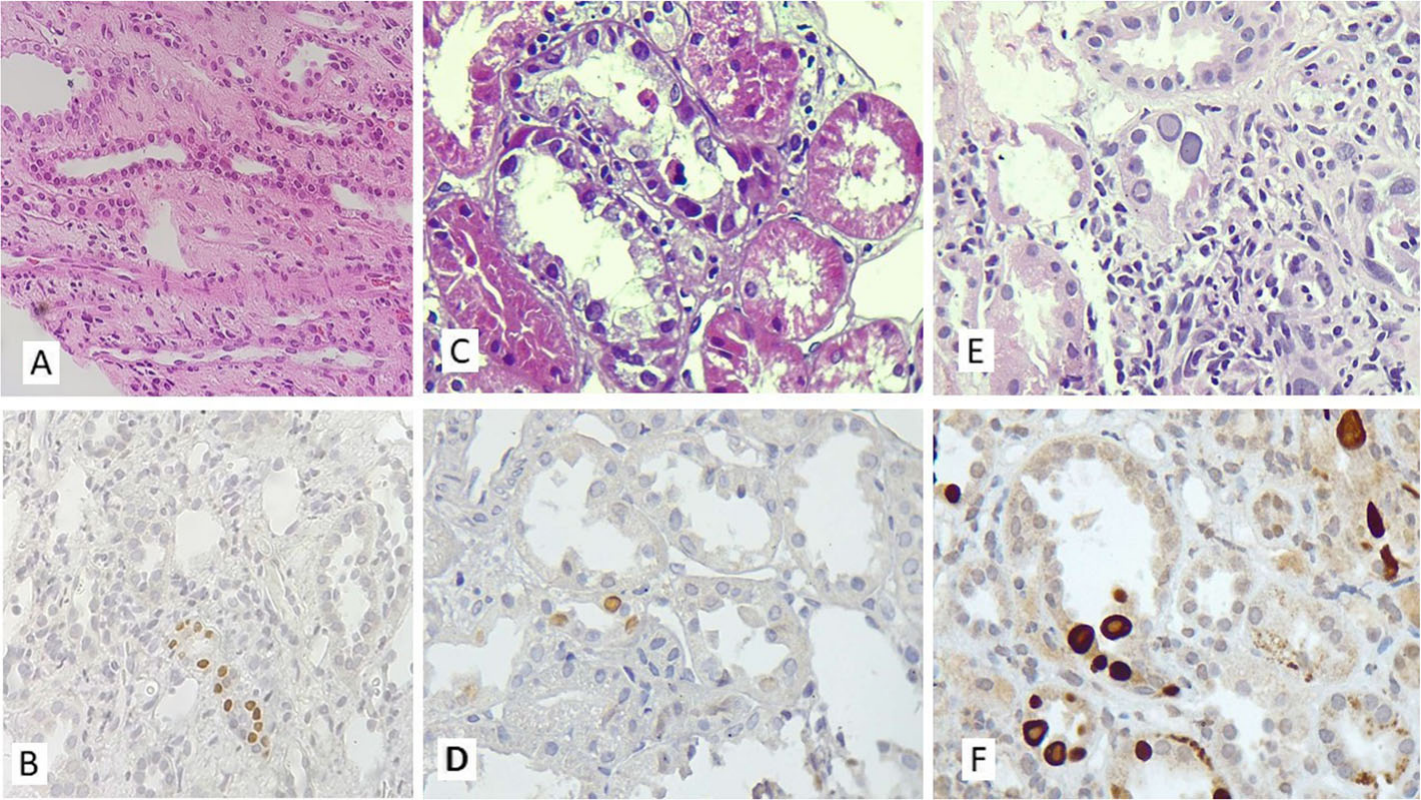
**a** Hematoxilin and Eosin stained sections shows medullary kidney tissue without significant pathologic finding (100X), **b** IHC study for SV40 reveal infection of some tubular epithelial cells (100X). **c** This biopsy specimen shows mild interstitial inflammation and tubular epithelial cells with suspicious atypical nuclei but not typical viral inclusion (400X). **d** IHC study (SV40) confirmed infection of epithelial cells with BK virus (400X). **e** Another sample shows typical basophilic ground-glass viral inclusions in tubular epithelial cells (400X) and **f** strong positive reaction for SV40 (400X)

In 261 cases, no obvious viral cytopathic effect in favor of PVBKN, was found. IHC study revealed 257 negative cases for viral infection, but 4 cases showed positive staining, indicative of early stage viral infection and nephropathy. Two of these biopsies were completely normal, two others revealed suspicious atypical nuclei (Fig. 1). In 14 patients, typical viral inclusions were identified, which were confirmed on IHC study. In this way, by using IHC study for all kidney biopsy samples, we found 257 true negative and 14 true positive cases. If we did not use routine IHC and only relied on histopathological findings, we would have missed 4 cases. Threfore, histopathological evaluation without IHC had 4 false negative cases. All of the cases with typical viral inclusions were positive on IHC. Therefore, there was no false positivity on histopathology (Table 1). Sensitivity, specifity, PPV and NPV were calculated using the following formula to compare the diagnostic accuracy of histopathological evaluation with and without routine IHC.

Sensitivity: True positive/ (true positive + false negative cases) = 14/ (18) = 77.7%.

Specifity: True negative/ (true negative + false positive cases) = 257/ (257) =100%.

PPV: True positive/ (True positive + false positive) = 14/ (14 + 0) =100%.

NPV: True negative/ (True negative + false negative) = 257/ (261) = 98.4%.

Of 275 participants in our study, PCR results were available for 90 cases. Eleven patients with definite diagnosis of BKVN, including those without typical inclusion bodies (four patients), had positive PCR test (Serum viral load more than 10^4^ copy/ml), while data was not available for other patients.

## Discussion

The frequency of PVBKN in patients with renal dysfunction after transplantation was 6.5%, in our study. The prevalence of PVBKN is variable among different populations. The overall prevalence is 1–10% [20]. Another study carried out by Ghafari et al. in 2008 in Iran, reported an incidence of 13.1% [7]. Routine screening of the patients based on well-established guidelines for early detection of virus activation and adjustment of immunosuppressive drugs has a great influence on incidence and outcome of PVBKN [21–23], which may not be regularly performed in all patients due to economic problems. Immunosuppressive regimens and genetic factors can also influence the susceptibility of patients to develop PVBKN. Rohn et.al suggested that a predisposition based on genetic polymorphism in HLA-E can identify the patients with increased risk of PVBKN [7, 9, 24].

At follow-up, 61.5% of PVBKN patients in our study had good kidney function after adjustment of immunosuppressive drugs, and 38.5% of them had graft loss. This is roughly similar to the global statistics of PVBKN in which 40–80% of kidney transplants result in graft loss.

The early accurate diagnosis of PVBKN in patients with kidney transplantation is a challenging issue. Quantitative real time PCR, and histopathological assessment of kidney biopsies have some limitations. Viral cytopathic effects including basophilic grand-glass nuclear inclusions are the characteristic findings for definite diagnosis of PVBKN [14]. However, any interstitial inflammation and tubulitis which cause regenerative nuclear changes in tubular epithelial cells or some other viral infections such as adenovirus can mimic PVBK infection. On the other hand, in early stage of the PVBKN, typical viral inclusion bodies cannot be detected in infected cells [14]. IHC or ISH assays for Polyomavirus antigens including SV40 can be confirmatory in these conditions. It is too important to diagnose early stage of PVBKN on kidney biopsy. So, we conducted this study to evaluate the significance of routine IHC testing for improvement of disease diagnosis in early stages. It should be noted that BK virus can be activated in the allograft tissue, early weeks after transplantations [2]. But early raised creatinine in this period is mainly because of ischemia or acute rejection. Therefore, we included patients with a new onset of increased creatinine, 2 weeks after transplantation in our study. As the result, among 18 definite cases with PVBKN, 14(77.7%) showed typical viral inclusion bodies on H&E slides. However, in 4(22.2%) of PVBKN patients, virus-infected cells were identified after IHC testing. Since most of these patients are stage 1, in the absence of concomitant pathologies, early diagnosis usually leads to appropriate treatment and a higher chance of recovery.

A study in the United Kingdom in 2008, reported that 2 of the 6 major transplant centers in London performed IHC for SV40 on all samples, and PVBKN detection rates were 4.9% in these centers in comparison with 1.6% detection rate in other centers [16, 25]. Isaac et al., performed IHC study to diagnose viral infection in different pathology specimens. Polyomavirus was detected in 22(14.3%) out of 154 samples. Most of the cases revealed typical viral inclusions. Four (18.1%) out of the 22 samples showed suspicious viral cytopathic effect and were confirmed by IHC [26]. In the most recent and largest cohort by Banff working group on definite cases of PVBKN, 19% of the biopsies did not show typical viral inclusion bodies and diagnosis was made after IHC study for SV40 [5]. White et al. emphasized that early diagnosis can improve outcome and advocated the policy of screening and routine IHC SV40 staining for all allograft biopsies [16, 25].

Drachenberg et al. evaluated 601 biopsy samples from 365 patients by routine light microscopy and IHC study for SV40. PVBKN was identified in 1.8% of the samples, all of them showing viral inclusion which were positive for SV40 immunostaining. They recommended ancillary IHC or in situ hybridization assays for confirmation of viral infection in cases with suspicious nuclear features, but not as a screening tool [27]. In contrast to that study, we had four patients without typical nuclear viral inclusions on light microscopy. Two were completely normal (Fig. 1a) and were diagnosed after IHC study (Fig. 1b). The prevalence of the disease which can affects the choice of a test for screening, was also much lower than our study.

Nickeleit et al. also suggest IHC, only for cases with suspicious histopathologic findings, presence of decoy cells on urine cytology or high level of serum viral load [14]. All of our patients with definitive PVBKN with or without nuclear inclusions, had a positive PCR test result. Although IHC study could be limited to the patients with suspicious nuclei and those with viremia or high level of viruria, but in practice, pathologist is not always informed about the PCR test result. Definite diagnosis is based on identification of viral infection in biopsy specimen and early diagnosis is crucial for appropriate treatment. Finally, PCR has some limitations. Although 10–30% of the recipients have viremia, PVBKN develops in about 2% [28]. Intra and inter-laboratory variation in quantitative PCR test results is well described [28] and the results should be interpreted with caution.

SV40 is an available marker in most surgical pathology laboratories with transplant biopsy samples. IHC study on paraffin blocks is an easy, rapid and non-expensive method. The cost of IHC study is comparable with PCR test. Enhancement of diagnostic accuracy, will reduce the risk of graft dysfunction and subsequent costs for additional treatments, dialysis or re-transplantation. Therefore, additional cost of staining is justifiable to perform screening on all biopsies, especially in the populations with higher prevalence of PVBKN and the patients who are not subjected to a routine BK screening program.

The lack of an available gold standard method for accurate determination of false negative and false positive results for different methods is the main limitation of most studies in this subject. Singh and colleagues suggested a non-invasive novel biomarker for assessment of PVBKN. It is based on the detection of three-dimensional polyomavirus aggregates in voided urine samples by negative staining electron microscopy. The presence of these urinary cast-like structures named “Haufen” has positive and negative predictive values over 90% by far more than other screening tests [29–32]. But this method is not available in most of the centers and we were not able to perform it for our patients. As many other previous studies, kidney biopsy histopathologic examination with IHC study was considered as the gold standard method, despite its limitations, in our study.

Due to low frequency of PVBKN, large multicenter studies are needed for the identification of the best screening test methods, accurate prevalence of the disease, risk factors of nephropathy development and progression.

## Conclusion

In our experience, routine IHC study for SV40 on all transplanted kidney biopsies of the patients with new onset of allograft dysfunction, can improve the sensitivity of PVBKN pathologic diagnosis. It is recommended to do especially in that populations with high prevalence of the disease and the patients who are not subjected to a routine BK screening program.

## Data Availability

The dataset used and/or analyzed during the current study available from the corresponding author on reasonable request.
